# The Stomach in Its Morbid States, &c. &c.

**Published:** 1838-07-01

**Authors:** 


The Stomach in its Morbid States, &c. &c. By *Langston Parker*, M.R.C. Surgeons, &c. 8vo. pp. 302. Longman and Co. 1838.

Many disorders of the stomach are not yet explored—and still more of them are mistaken or mismanaged. This centre of sympathies is exposed, more than any other organ to a variety of deleterious agencies, and the disorders thence resulting are reflected from part to part so obscurely and mysteriously, that the practitioner is bewildered in the maze of anomalous and unaccountable phenomena. In this dilemma he is often induced to abandon the attempt to unravel the primary source of the multifarious symptoms, and direct his attention merely to the most prominent and pressing. The work before us is one of great merit—not so much on account of its originality, as of the care and judgment which Mr. P. has exercised in selecting from various Continental physicians and pathologists, a mass of facts and opinions elucidating numerous disorders of the stomach.

The work is divided into thirteen chapters, and embraces an immense number of divisions and subdivisions. The author considers that the stomach presents two grand or primitive morbid states—one characterized by lesion of its sensibility—the other of lesions of its circulation, comprising that great variety of affections called and treated inflammatory.

1. The first chapter takes up, contrary we think to the order of Nature, the inflammatory affections, beginning with mere vascular congestion, or morbid turgescence of the vessels in the mucous coat, interfering with the function of digestion, and if unchecked, going on to inflammation. "This morbid fulness of blood in the mucous coat results from *irritation*, generally prolonged and frequently repeated." Thus the nervous system of the organ is acknowledged by the author, to be first implicated even in this the lightest shade of vascular derangement. These irritations are chiefly dietetic, but may come on in consequence of several diseases. "Persons thus affected are on the brink of a serious disease, but not in it." The symptoms vary from mere sense of distention after food, up to actual vomiting, thirst, pain, fever and head-ache. Many appellations have been given to this state of things;—as gastric irritation, indigestion, dyspepsia, and many foreign names of difficult pronunciation. Broussais, and the disciples of his school, maintain that this is veritable *gastritis*, and more than temporary or evanescent congestion; but to this doctrine our author demurs, and so do we. The question is not easily decided, as we have few opportunities of *post mortem* examinations—and even if a man died of another disease, but labouring under this form of indigestion, the state of the stomach, after death, could bring no conviction to the mind of a cautious investigator.

Be this as it may, our author thinks that this congestive state, if neglected, may terminate in various ways—in true gastritis, or diseases, from sympathy of the liver, brain, heart, and lining of the bronchiæ. This vascular congestion of the stomach is a common attendant upon all, or almost all, inflammatory and pyrexial affections in other parts of the body, and ought to be borne in mind when we are prescribing medicines for them. This same gastric condition is also very often set up at the termination of acute diseases, and throws them back into relapses from convalescence. It is usually the result of too much food, when the craving appetite returns. Most eruptive fevers present this state of stomach at the beginning.

### CHAPTER THE SECOND.—ANEMIA.

Our author maintains that this condition of stomach—the very reverse of the former—" presents all the symptoms depending on vascular irritation —namely, epigastric tenderness, and pulsation, vomiting, distention after food, &c." He adduces some cases in illustration. We are tempted to introduce one of these cases.

" A lady, aged thirty, miscarried in the third month of her pregnancy, at which time she lost much blood. At the present time, two months after the abortion, she is labouring under the following train of symptoms:—Great pain in the epigastrium, aggravated by pressure, and accompanied by strong pulsation in this region. Fulness, pain, and distention after meals, with nausea, occasional vomiting, palpitations, and inactive bowels. A medical practitioner, supposing these symptoms were dependent upon some inflammatory affection, had ordered leeches to the stomach, which had aggravated all the symptoms. The pil. aloes, assafœt. et saponis, was ordered subsequently, to regulate the bowels, and chalybeates were freely given. Under this plan, the pain, tenderness, pulsations, vomiting, and distention disappeared, and the patient recovered her usual health." 13.

In the above and in similar cases, the disturbance of function must be mainly dependent on lesion of the gastric nerves, and it shews us how difficult it is, sometimes, to discriminate morbid sensibility from congestion or chronic gastritis. In such cases, the attendant and antecedent circumstances will guide us—and the effect of remedies will, at all events, unravel the mystery. The following opinion is quoted from Andral.

" ' It is a law in pathology that, in every organ, the diminution of the quantity of blood which it should contain in a healthy state, produces functional disturbances, as well as the presence of an excessive quantity of blood; and what is more, in both cases these functional disturbances are precisely similar.' "* 15.

But we imagine that another explanation of these phenomena might be attempted, if not sustained. Bleed an animal to death, and the vessels of the brain will be found gorged with blood, or actually burst. In such cases Nature seems to throw all the blood she can preserve upon the most important organ or organs of the body; and we are by no means certain that such is not the case as regards the stomach, in hæmorrhages and other losses.

### CHAPTER THE THIRD.

This Chapter is dedicated to a " general review of the symptoms and sympathies dependent on vascular irritation of the stomach." This review is thrown into different heads—namely, the state of the tongue—the state of

* " Andral. Clinique Medicale."

the epigastric and hypochondriac regions—the organs of respiration—pulse and heart—brain and senses—local pains. These are all discussed in fourteen pages of the work. 1. *Tongue.* Though the state of this organ is very variable, and bears no strict relation to the degree of gastric disorder, yet it generally presents an appearance of contraction, dryness, coat in the centre, redness at the tip and edges, erection of the papillæ. But when the gastric sensibility is very morbid, the tongue will often be coated throughout, with vividly red papillæ springing up through it, there being merely a red line at the tip and edges. Under such conditions we will frequently find great despondency, languor, epigastric uneasiness of an indefinable kind, especially on pressure. A third state is the beef-steak tongue, and where the almost total surface is red and smooth, denuded as it were, of its papillæ. In this kind of tongue there are often two white streaks, one on each side, running parallel with the raphæ—indicating serious disorder of the stomach. Mr. Parker thinks it indicates " a thickened or scirrhous state" of the coats of the stomach ; but we can assure him, from no very limited range of experience, that this prognosis or diagnosis is much too grave. We agree with Mr. Parker, however, that with such a state of tongue, the epigastric region should be carefully examined—for, as he justly observes, the tongue itself, isolated from other phenomena, affords no criterion of the condition of the stomach.

2. *Epigastrium.* When the regions of the stomach, liver, duodenum, and spleen are carefully examined, even at the commencement of inflammatory irritation, some physical signs will generally be observed. Sometimes there are fixed pains in different points of the epigastrium or hypochondria. We recommend the following sentiment to our friend Dr. W. Philip.

" The state of sensibility in the centre of the epigastrium, which corresponds to the situation of the great nervous ganglia of the abdomen, is extremely deceptive, since most persons are sensible to moderate pressure in this situation." 22.

*Fulness* in epigastrio is more significant—and in advanced stages is converted into hardness and dulness on percussion. Inflation, however, from flatulence, will render these parts sonorous, even where there is considerable thickening. The temperature of the epigastrium is often increased—and is not uncommon; and constipation is usually complained of, though *irregularity* of bowels is by no means infrequent. Pulsations and tremulous motions in the epigastrium are common attendants on incipient gastritis—sometimes evanescent, sometimes more permanent. Often they are only perceptible by the patient, and not by the practitioner.

3. *Organs of Respiration.* In this section our experience is at variance with our author. He avers that " no system is more commonly affected from inflammation in the stomach than the organs of respiration." We appeal to every practitioner of experience whether even in the most acute gastritis, the lungs are commonly affected ? It is quite clear that Mr. Parker has been brought up in the French school, and that more of his opinions have been founded on the lectures of the Parisian physicians than on his own clinical observations. Not a day passes in which we do not see the deplorable consequences of attributing *cough* to the stomach. Let our readers ponder on the following passage.

"Several symptoms of disease in the stomach are to be found in the organs of respiration. The first is a short dry cough, which has been denominated, by the French, *toux gastrique;* sometimes this is the only symptom, but it may be accompanied by expectoration of frothy, bloody, heavy, discoloured mucus, accompanied by pains in the sternum, epigastrium, hypochondria, or some points of the thoracic parietes." 24.

Why if such symptoms as the above are to be referred to the stomach, we may save ourselves the trouble, in future, of all thoracic investigations. But our author informs us that, in these cases, the stethoscope will tell us there is nothing affecting the integrity of the lungs or their membranes! What! is frothy, bloody, heavy, discoloured mucous expectoration to be present, and no affection of the lungs or their membranes in existence? If these symptoms exist, and the stethoscope only tells of sympathy with the stomach, then auscultation is a curse, and not an important auxiliary in medicine. It is an ignis fatuus leading the practitioner into the most fatal of all errors! But even granting to our author that the pulmonic affection has been brought on by gastric disorder, we maintain that all thoughts of treating the *latter* must be abandoned till we have subdued the pulmonary affection.

4. *The Pulse and Heart.* On this point also, we cannot run parallel with our author, and he will hardly accuse us of entire want of experience. The following passage will shew that our author is occasionally led from the sober path of personal investigation by the Continental pathologists.

"The heart may be affected by irritation in the stomach in many ways; by mere increased impulse, by occasional intermissions of its pulsations, or by irregular and tumultuous motions. In some instances, an actual physical sign of disease may be present, and after death the heart be found perfectly healthy. Cruveilhier has recorded a case of this nature, where the action of the heart and the morbid sound it emitted was dependent upon stomach disease. I have recorded another, which will be found in a subsequent part of this volume. *The pulse is also liable to occasional variations, which are sometimes independent of the action of the heart. These symptoms are manifested in a double or triple motion of the artery to each contraction of the ventricle.*" 26.

Now we appeal to every auscultator in these kingdoms whether the passage marked in Italics by us, does not involve a physical impossibility. The pulse acting two or three times to one contraction of the ventricle!! It is an absolute impossibility; and we would just as soon believe that the dead man called out from his grave to the people walking over his tomb. Every one knows that the ventricle will often act, for many hours together, as before death and in cholera, without any perceptible pulses; but that the pulse should be in activity while the heart is quiescent, is a solecism in physiology. It would be a miracle—and miracles have ceased. If Mr. Parker should ever meet with such a case, and shew it to us, we will engage to pay him down one hundred guineas for the curiosity. Mr. Parker runs into another extreme, though not involving an utter impossibility, in the following observation.

"I have recorded one or two cases where the only symptoms of *diseased heart* were pain and tenderness in the epigastrium, nausea, distention, and weight after eating, and occasional vomiting." 27.

Now if a *disease* of the heart can exist without any symptom, but those dependent on the stomach, our auscultators may throw away their stethoscopes! The plain fact is, that disorders of the stomach will often interfere with regularity of function in the heart. Thus intermissions of the pulse, temporary palpitations, &c. are many times produced by flatulence, acidity, and other affections of the stomach; but we confess that we have never met with an instance where *disease* of the heart gave no indication of its existence except through the medium of the stomach.

5. *The Brain and Senses.*—That the brain sympathises largely with the stomach, no man can doubt; but even here we see daily errors committed by too credulously referring sensorial affections to the stomach. It is a very serious mistake too, when it is committed. We agree with Mr. Parker that stupor, giddiness, and confusion of thought are often the effects of gastric derangements, and that despondency, from gastric sympathy, is still more frequent; but we ought to investigate the case very cautiously before we come to the conclusion that the above symptoms, as well as head-aches, loss of memory, muscæ volitantes, mania, &c. &c. are merely symptomatic of stomach disorder. The mistake may be fatal, and the responsibility is fearful. Our own practice is to direct the chief attention to the head, at the same time that due care is taken not to injure the stomach—and whenever there is a doubt on the subject, to decide in favour of cerebral affection as the primitive or important malady.

6. *Local Pains.*—These are various in kind as well as in situation. There is not a part of the body that may not be occasionally the seat of these sympathetic pains. Young practitioners are constantly mistaking them for inflammations, and applying leeches unnecessarily. When in the pleura or intercostal muscles, they are often treated as pleurisy or pneumonia. Independently of the stethoscope the non-pyrexial condition of the pulse, the skin, and various indications, will apprize the observant practitioner of the nature of these local affections.

### Chap. IV.—Confirmed Inflammatory Affections of Stomach.

The transition from a mere hyperemic to an inflammatory state of stomach is a fine theme for the lecturer amongst his gaping pupils—but the practitioner at the bedside of sickness knows the fallacy of nine-tenths of the criteria laid down by teachers and authors. The state of gastric congestion —and still more that of morbid sensibility of the gastric and duodenal nerves presents, in numerous cases, far more active and prominent symptoms than the state of actual chronic inflammation, even with hypertrophy or ramollissement of the coats of the organ. In this last condition we have often no more than constipation of bowels, dull pain in the epigastrium, unhealthy aspect, depression of spirits, anorexia, flatulence, and slight emaciation. The tongue does not afford any positive criterion. "We may have a clear tongue with a diseased stomach—a diseased tongue with a healthy stomach—or disease co-existing in both organs, but independent of each other." The vermillion redness of the papillæ about the tip and edges is, perhaps, as certain a criterion, as far as the tongue is concerned, as we have of chronic gastritis; but it is not upon one, two, or three symptoms we can depend.

In respect to the symptoms of chronic gastritis, as gathered from the function of the organ itself, they are various—loss of appetite, impaired, and slow and painful digestion, with distention, nausea, and a host of anomalous feelings that defy description, and which cannot be accounted for, even with all the advantage of excito-motory doctrines. Numerous cases are detailed, and a large mass of observations are drawn from foreign authors, especially Andral and Broussais, in illustration of this part of the subject. From the following quotation, it would appear that our author has been an *interne* of some of the Parisian hospitals, and that his experience and doctrines have been drawn almost entirely from such sources, under the guidance of our Gallic brethren.

"I have had the charge of several patients in the latter stages of gastric diseases, who have been able distinctly to trace the commencement of their complaints. These have seldom commenced before the age of twenty-five, at the periods when they had begun the habitual use of a fuller and more stimulating diet than that of the earlier periods of life. The symptoms with which they were first affected were those of simple indigestion, in its various forms of pain or distention after food, nausea, or vomiting. These have ceased at intervals, have been relieved by various plans of treatment, but have shown a disposition to recur at longer or shorter intervals from dietetic errors or excesses, or from other causes, in more aggravated and obstinate forms than those in which they first made their appearance, and accompanied by sympathetic irritations in the head, heart, liver, or lungs, exhibited in the forms of giddiness, palpitations, jaundice, or cough.

On examining the bodies of such patients after death, what has been the condition of the organs exhibiting these symptoms during life? In the stomach, changes of colour or consistence, ulcerations or vegetations; in the brain, thickening of its membranes, effusions, increased determinations of blood; in the heart, alterations of its internal or investing membranes, or, what is more common, of its muscular structure; in the lungs, congested or inflamed states of the bronchial mucous surfaces, or of the lungs themselves; and in the hepatic system, a diseased condition of the veins or substance of the liver, alterations of colour or consistence, and various morbid states of the bile, the gall-bladder, and the excretory passages of the bile." 47.

The foregoing pathological phenomena present no very cheering prospect to the dyspeptic invalid, whether in or out of the profession. But fortunately they are rarely the result of indigestion; and when we find such multiplied lesions, *post mortem*, in the three great cavities of the body, who can affirm that they all sprung from chronic gastritis? Broussais and his disciples will answer in the affirmative; but the thing is too preposterous to gain credence from the sober practitioners on this side of the channel.

### CHAP. V.—MORBID SENSIBILITY OF THE NERVES OF THE STOMACH.

"I have shewn, in the preceding chapter, that the characters of pain attendant upon inflammatory conditions of the stomach are extremely variable, being sometimes obscure, at others violent, bearing no strict relation to the character or degree of that inflammation upon which they depend, or with which they are associated. There are many diseases of the stomach which consist in a purely morbid state of the sensibility of the gastric nerves, without the association of any inflammatory action; these have been well described by Dr. James Johnson, in his work *On the Morbid Sensibility of the Stomach and Bowels*, and into their primary forms I consider it unnecessary to enter, referring my readers to that book for information on these points.

M. Barras has produced a work of some merit, entitled *Sur les Gastralgies et les Entéralgies*, or Nervous Diseases of the Stomach and Intestines. A careful perusal of the work will, however, shew that many of the cases detailed in it were evidently inflammatory in their commencement, and only rendered purely nervous by the pernicious system of large local depletions, adopted by most of the French physicians of the physiologic school, acting upon the aphorism of Broussais, that the greater part of all indigestions, in whatever form they are exhibited, are due to a chronic inflammation of the mucous coat of the stomach. M. Barras maintains, merely in opposition to M. Broussais, that the greater part of these affections are of a nervous kind, and dependent altogether upon certain and varied states of depraved sensation in the gastric and intestinal nerves; and to these causes he refers the group of dyspeptic symptoms of pain, cramp, spasm, chronic vomiting, morbidly increased, depraved, or defective appetite, and other symptoms of this kind.

The truth, however, rests neither with M. Broussais nor M. Barras, but between the two." 53.

This we believe; but we confess that we are far more inclined to side with Barras than with Broussais; and long and painful experience has convinced us that, however *gastritis* may become mixed up with *gastralgia*, in the progress of indigestion, the latter takes precedence in nineteen cases out of twenty. Examples are quoted by Mr. Parker illustrating the form under consideration. This form (morbid sensibility) may be either acute or chronic, and attended with a host of anomalous sympathetic affections of other and distant parts. Emaciation is a common consequence—partly from the patient's inability to take food, and partly from the miserable feelings he is destined to endure. Numerous cases are collected by our author from various sources, as examples of MORBID SENSIBILITY, for which we must refer to the work itself. If the following refinements of the physiological school of young France and Germany should enable any of our readers to discriminate the minute and often evanescent shades that distinguish, or rather confound, inflammatory with nervous affections of the stomach, we shall be extremely gratified.

"It is of the greatest consequence, as regards the treatment of diseases of the stomach, both in their primary and advanced stages, to ascertain whether the symptoms they exhibit are dependent upon nervous or vascular irritation, whether the affection be unaccompanied by inflammatory action, or whether the nervous symptoms which are manifested during the progress of the disease depend upon inflammation or not. I believe that, in a great majority of instances, an increased fulness of blood in the mucous membrane of the stomach is present, at least during the paroxysm of the attack; yet we must be extremely cautious how we take such an opinion as the sole basis of our treatment. The degree of nervous irritation exhibited, whether this consist in actual pain, in mental despondency or irritation, in anomalous feelings attended by various exalted or diminished states of the sensibility, must be examined in relation to the actual state of vascular excitement with which they are or are not accompanied, and the treatment proportioned accordingly. The relative proportions which these two states of excitement bear to each other, and the degree in which each is developed, must form the basis of all rational treatment, both medicinal and dietetic. Occasionally the symptoms dependent upon an inflamed state of the mucous coat of the bowels are confined almost entirely to those which are called nervous—*i. e.*, phenomena which are attributed entirely to the nervous system alone—are those merely which are found as indicative of inflammatory disease. I shall take the table arranged by Jolly for the basis of the distinctions between these two states."

TABLE.

| Symptoms of the Nervous Affections of the Stomach. | Symptoms of the Inflammatory Affections of the Stomach. |
|---|---|
| *Pain.**—Acute, tearing<br>Intermittent<br>Diminished by pressure<br>And by taking food<br>More frequently coming on in the morning | *Pain.**—Dull, obscure<br>Constant<br>Augmented by pressure<br>And by food<br>Increasing towards the evening |
| *Tongue.*—Sometimes coated†<br>Broad<br>Clean | *Tongue.*—Almost always red†<br>Contracted<br>Thickly coated |
| *Appetite.*—Morbidly increased‡<br>Depraved<br>Wish for high-seasoned meats and alcoholic drinks | *Appetite.*—Wanting‡<br>Never depraved<br>Aversion to both |
| *Taste.*—Metallic<br>Acid | *Taste.*—Bitter<br>Clammy |
| *Vomiting* of mucous discharges | *Vomiting* of food |
| *Alternations of Heat and Cold* in the abdomen | *Constant Heat* |
| *Thirst.*—Not increased<br>Wish for drinks sometimes hot, at others cold | *Thirst.*—Increased<br>Constant desire for cold drinks |
| *Constipation.*—Frequent‖ | *Diarrhœa.*—Frequent‖ |

* The two characters of pain described by Jolly cannot be depended upon as shewing any certain distinction between inflammatory and nervous disease of the stomach, that of the nervous kind is so constantly associated with, produces, and succeeds to, partial inflammations of the mucous coat.

† The state of the tongue is infinitely variable in the different forms of disease. The observations of Louis and Andral, and my own cases, shew that it may remain clean in aggravated forms of inflammatory disease, even where change of structure has been produced. It may be foul, and even aphthous, where the gastric inflammation is not urgent or well-marked.

‡ Boulimia, or morbidly increased appetite, is a symptom commonly attendant on an inflamed condition of the mucous coat of the stomach. Total loss of appetite may also be present, and, again, this function may remain unimpaired. We see how uncertain are all these rational signs of disease. A remarkable case of morbidly increased appetite, attendant upon cancer of the stomach, is detailed in the *Lancet* of October 1, 1836.

‖ Constipation is a symptom almost invariably accompanying both forms of disease. Diarrhœa is certainly present in some instances of the inflammatory affections, even in the commencement of disease; in the advanced stages, where complaint has extended to the bowels, it is of more frequent occurrence.

| Symptoms of the Nervous Affections of the Stomach. | Symptoms of the Inflammatory Affections of the Stomach. |
|---|---|
| *Stools.*—Natural<br>Not offensive | *Evacuations.*—Bilious, mucous, or bloody<br>offensive |
| *Pulsations in the Epigastrium.*—Intermittent<br>Not synchronous with those of the heart | *Pulsations in the Epigastrium.*—Natural, continual<br>Synchronous with the heart |
| *No Fever*<br>Or intermittent | *Fever.*—Frequent<br>Continued |
| *Increase of Disease.*—Early in the day | *Increase of Disease.*—In the evening |
| *Urine.*—Clear*<br>abundant | *Urine.*—High-coloured*<br>Scanty |
| *Heat of Skin.*—Natural | *Heat of Skin.*—Augmented |
| *No Progressive Emaciation* | *Progressive Emaciation.* |
| *Physiognomy.*—Natural | *Face.*—Pale, sallow, or sunk and anxious |
| *Temper.*—Morose, fearful, irritable | *Temper.*—Little altered |
| *Diagnostic.*—Sometimes obscure | *Diagnostic.*—More manifest |
| *Prognostic.*—Less dangerous | *Prognostic.*—More dangerous |
| *Anatomical Characters.*—Equivocal, or altogether wanting | *Anatomical Characters.*—Constant, but varied |

If we had made the following confession, there would have been no end to the criticisms heaped upon us by the physiological refiners, and the German dreamers on this side of the Channel.

"The symptoms of vascular and nervous irritation of the stomach are sometimes so similar that the most experienced practitioner in diseases of this kind is occasionally at a loss to decide upon their precise pathologic character." 76.

"*Sometimes!*" For sometimes read *very often*, and you will be much nearer the truth.

The sixth chapter is "on Affections of the Stomach characterized by Morbid States of its Secretions." This, Mr. Parker thinks, may be a *primary* morbid state, resembling one or both of the two primary morbid conditions already described, but differing in its pathological character—and aggravated by the remedies employed for either of the other, and too often similar, affections. Here it is evident that we are led on from one refinement to another, till at last the practitioner of half a century's experience and observation, is completely bewildered, and gives himself up to despair. A long life devoted exclusively to the management of gastric affections would be a great deal too short to study the refinements, not of our author, but of the French school where he has imbibed his doctrines.

This form of the complaint is the "embarras gastrique" of some of our

* Dézeimeris has related some cases where diabetes appeared dependent upon chronic gastritis, at least the cure of the gastritis completely removed the diabetes. Andral has also detailed a case where diabetes coincided with chronic gastritis; the former disease was removed by curing the latter.—*Clinique Medicale*, by Spillan, p. 869.

Gallic neighbours—the disorder of the "chylopoietic organs" of Abernethy, Curry, Hamilton, and half the practitioners of England—the first shade of gastro-enteric inflammation of Broussais and all his disciples.

"In this affection, as I have before said, a morbid condition of the secretions is the predominant feature of the disease. Professor Recamier has detailed accounts of the dissection of several subjects who have sunk from aggravated diseases of this character. He found, in these cases, the liver pale and voluminous, the gall-bladder full of black bile, which was extravasated in large quantities in the duodenum, jejunum, and ileum; the stomach and duodenum were likewise coated with thick viscid secretions, under which the mucous membrane did not present the least trace of inflammation or congestion. Andral has also described a disease under the name of gastrorrhœa, in which he considers this state of the secretions, as a predominant and primitive feature of disease, to be as well established as any other point of pathology." 80.

Our author tells us that the symptoms of this form resemble, in some manner, those of hyperemia, or active congestion of the gastric mucous membrane—and may be divided into those phenomena which refer to the stomach itself—and those which include various sympathetic affections of other organs. "The tongue is generally broad, rather pale and moist, not contracted, and without increased redness at its point and edges. The papillæ are not elevated, enlarged, or vivid in their appearance; the coating is thick, and of a dirty white or yellowish colour. The epigastric region is full, but indolent—hardly sensible or tender under pressure, unless the disease be of long standing."

"As a disordered condition of the secretions of the stomach may be combined with congestion or inflammation, in some instances, so may it in others be associated with a variety of nervous symptoms, which are totally different from those indicating increased determination of blood, either to the stomach or parts sympathetically affected." 82.

By this time, we imagine our readers, as well as ourselves, are involved in a pretty considerable labyrinth of perplexities—and if they can see their way clearly out of the wood, it is more than we can! Numerous cases are cited from authors, and related from Mr. Parker's own observation, for which we must refer to the work.

We are quite willing to admit the frequency of this form of gastric disorder—indeed of its being more frequent than any other form. But we would ask the author, and our experienced readers whether in this, as well as in *all* other shades of the complaint, the nerves of the stomach are not primarily implicated in the disordered secretions? Is there ever a *morbid secretion* without antecedent *morbid sensibility?* We do not believe there is. Every grade and shade of moral impressions of a sombre cast, will load the tongue and derange the secretions. And through what medium? The nerves most assuredly.

### CHAP. VII.—ON THE INFLUENCE OF THE STOMACH UPON OTHER ORGANS.

1. *On the Liver.* There is no doubt this organ is amongst the first to get disordered in function through sympathy with the stomach. The hepatic secretion is that which gives the most prominent and appreciable character to the alvine evacuations, and hence we say the liver is torpid when the motions are pale—that it is inordinately active, when the egesta are of an orange colour and copious—that the biliary secretion is vitiated, when the motions are black, green, olive, and other colours, with detestable fætor, &c. The misery which these poisonous secretions occasion in the animal economy are indescribable—and undescribed. The vitiated bile which is poured into an irritable—a morbidly sensitive duodenum, has caused many a suicide, and induces torments which no language can portray! But these vitiated biliary secretions derange the whole of the innumerable secreting glands that stud the alimentary canal from the stomach to the rectum, augmenting the wretched feeling of the patient, and spreading the circle of sympathies to every part of the body, and every faculty of the mind. These consequences are not sufficiently adverted to by the Continental pathologists. They are perpetually in search of scalpel phenomena, and when they find them after death, they throw little light on the original disorder! The French therapeia is very defective in this class of maladies. The ghosts of gastritis and enteritis are so perpetually before their eyes, that purgation is quite out of the question—and even the mildest aperients are looked upon with a degree of dread little short of that with which a hydrophobic patient contemplates a pail of water! Now we do not recommend drastic purgatives in these disordered alvine secretions, though they are often necessary; but we do maintain that, unless these poisonous matters are daily carried off, we shall protract the sufferings of our patient and render more difficult the cure of the disease.

2. *The Lungs.* Upon this subject we are at variance with our author, and with the French physicians generally. We know, by mournful experience, that it would be fortunate if the gastro-pulmonic sympathy were blotted out of the catalogue. Granting that coughs, pneumoniæ, and pleurisies are *always* (and surely this is going far enough) secondary to gastro-enterité, it matters not one jot. The disease is to be attacked in its new seat, just as though there never had been gastritis or enteritis at all. But the new doctrine denies, or at all events, neglects this practical rule, and treats pneumonia, or bronchitis, by remedies directed to the gastric affection, of which it is supposed to be symptomatic. Thus the time is often lost for quelling the pulmonic inflammation, while trifling remedies are prescribed for the gastro-enterité! Thus scarcely a day passes in which we do not see patients labouring under cough, quick pulse, dry skin, scanty urine, evening fever, &c., who have been ordered by the physicians to eat nothing but animal food in the most concentrated form—because their cough was *stomach* cough, dependent on indigestion—and because mutton is good for dyspepsia! On examination of the chest, disease of the lungs or membranes are easily discovered—and the mischief is generally irremediable. Such are the effects of the doctrine of stomach-cough. The mistake in the opposite direction can do no possible injury.

3. *The Heart.* We agree with Mr. Parker that the action of the heart is influenced in two ways by disorder of the stomach—by nervous sympathy, and by mechanical pressure. The action of the heart is always increased by food, independently of distention; but the presence of air or acid in weak stomachs will often cause intermissions of the pulse, and a sense of fluctuation about the heart, which alarms both patient and physician till the real cause is known. Auscultation now clears up the doubt at once.

4. *The Brain.* The vicinity of the ganglia and plexuses in the epigastric region, and the connexion of these with the brain itself, may easily account for a strong chain of sympathies between the stomach and the head. But independent of this, the great sympathetic and par vagum keep up such a direct intercourse between the two organs, that we need not wonder at the fact that, of all organs the brain is most frequently affected sympathetically by disorders of the stomach.

"It is difficult (says Dr. Johnson) to say which is the organ or part that is most intimately linked in sympathy with the stomach and liver. I should say, however, that the brain, as the common sensory, to which all sensations are ultimately referred, is the first to sympathise with disorder of the abdominal organs. Pain in some part of the head is a very common symptom in this class of disorders; but the *functions* of the brain are affected in a great variety of ways—especially its *intellectual* functions. Confusion of thought, unsteadiness of the mind, irritability of the temper, defect of the memory, fickleness of disposition, and many other phenomena which are little suspected of corporeal origin, shew themselves infinitely more often than pain, deafness, vertigo, defect of vision, or affections of mere sensation. The former gradually rise into gusts of passion, fits of despondency, brooding melancholy, permanent irascibility, and still higher grades of intellectual disturbance, till, as sometimes happens, the point of temporary alienation is reached, and suicide terminates the scene. Those functional disturbances of the brain, however, which are evinced in the form of mental phenomena, are very common in *morbid sensibility* of the gastric and intestinal nerves, where the usual symptoms of indigestion and hepatic derangement are almost entirely wanting, and these will be more distinctly alluded to hereafter. In unequivocal disorder of the digestive organs, the affections of sensation about the head most engage the patient's attention. Pains of various kinds, not seldom remittent or intermittent, are felt in different parts of the scalp, about the face, or deep in the head. When purely sympathetic of stomach disorder, they are more frequently in some particular part, than in the head generally, and assimilate in their nature to tic douloureux. Indeed, I have no doubt that this dreadful disease is, in many cases, caused by irritation of the visceral nerves—and the cures which have been performed by alterative and aperient medicines, and especially by the carbonate of iron (which removes the morbid sensibility of the nerves), confirm this opinion."*

The 8th chapter is a long one, tracing the influence of stomach disorder on the origin, progress, and termination of diseases of the liver—which chapter branches out into numerous sections, tracing, or retracing the influence of hepatic disease on various organs and functions. These we find it impossible to analyze—and we must again and again say that our author has become so impregnated with German minuteness as greatly to lessen the value of his work among the busy practitioners of this country. The numerous cases which Mr. Parker has collected from various authors, and related from his own experience, may be perused with much advantage by all classes of readers. There is a section on the influence of gastric affec-

* Essay on Indigestion, 9th Ed., p. 42-3.

tions in the formation of biliary calculi, which is curious. That we know very little of the causes of these troublesome concretions is confessed by all. They are, as every one knows, very often attended, perhaps preceded by dyspeptic affections; but it is rather hazardous to infer that these last are the causes of the former. There can be little doubt, however, that some derangement of the biliary secretion itself precedes the formation of biliary calculi. Assuming that all disorders of the stomach occasion disordered action in the liver, Mr. P. goes on to say that—

"In most persons dying from gastric disease, or from other diseases in which there is a serious complication of morbid conditions of the stomach, we find the contents of the gall-bladder altered in their character, and very commonly the lining membrane of this organ itself inflamed, softened, or otherwise diseased. In these states the contents of the gall-bladder are generally of extreme viscidity; the bile is black, resinous, adhesive, much thicker than in its natural state, and of a deep-black colour. I have noticed this condition of the bile in almost every instance I have examined after death from gastro-hepatic disease. Similar facts have been noticed by Portal and others." 162.

Broussais attributes the formation of biliary calculi to an inflamed condition of the liver and gall-bladder. Several cases are detailed by Mr. Parker in support of the doctrine of the gastric origin of these concretions.

The 9th chapter is occupied with considerations on the gastric origin of dropsies. Having shewn that gastritis "commonly precedes disease in the liver and heart, and, in a great majority of instances, accompanies both," he thus connects the effusion of serum in the peritoneal cavity with original phlogosis of the mucous membrane of the stomach. By pursuing this plan we shall very soon arrive at the conclusions of the celebrated Abernethy—that all diseases began in the stomach.

We have alluded to the opinion of our author, that the pulse may beat twice to one contraction of the left ventricle. The following is one of the cases in support of this position.

"A young lady, aged eight, after the disappearance of the eruption of scarlatina, was seized with sickness and constant vomiting of food, with pain and tenderness in the epigastrium: the bowels were constipated, the papillæ of the tongue enlarged and intensely red. The pulse was frequent, and had a double beat to each systole of the heart, two pulsations of the artery at the wrist to each single contraction of the left ventricle of the heart. 185.

We have no right to dispute a fact, when related by a credible witness; nor do we suppose that Mr. Parker states here anything that he does not firmly believe; but we must take the liberty to suppose that he was deceived in cases like the above, because it is just as impossible for the artery to beat twice for one contraction of the ventricle, as it would be for a mass of ice to form in the centre of a cauldron of boiling water. Yet Mr. Parker has related six or seven cases of this kind, and avers that he has seen instances where there were three pulsations at the wrist to one ventricular contraction! We would simply ask our author what is the *cause* of the pulse at the wrist? If Mr. P. maintains that it is *not* owing to a jet of blood thrown out of the ventricle, why then we drop the argument. If he acknowledges the causation which we have mentioned, then—"sublata causa tollitur effectus." However, we will not dwell longer on trifles like these, where there is so much to praise in the work under our notice.

We have considerably exceeded our prescribed limits, and must pass over a very long chapter, and several sections on the influence which disordered stomach exerts on diseases of the brain—and in which a great mass of research and personal observation is made to bear on this important topic.

The last chapter (XIII.) is on the treatment of disorders and diseases of the stomach, and occupies only twenty out of 300 pages. This is in keeping with the rest of Mr. Parker's work, which is founded on the French models—all, or almost all, physiology and pathology—very little on therapeutics. With this we do not find fault—quite the contrary. It shews a love of science, and not a tendency to quackery—too often apparent in works of this description. If we were well acquainted with the nature and causes of diseases, we could have but little difficulty in suggesting the treatment of them. Superficial writers dwell largely on the therapeutics, without the slightest knowledge of etiology and pathology !

Our author divides the treatment into dietetic and medicinal. "Of the former enough has been said in the writings of Paris, Johnson, Philip, Abernethy and others, to render a recurrence to it here unnecessary." The medicinal treatment must be suited to "the particular group of symptoms manifested by different individuals." On this plan or principle, he distributes the symptoms into several classes, taking the predominant symptoms which demand chief attention as the type of disease in the class to which it belongs. We shall glance at the principal classes or groups.

1. *Pain and Constipation.*—In such states Mr. Parker condemns drastic and cold saline medicines—and considers leeches and blisters as of little use. He relates a case to exemplify his treatment. A lady had suffered from pain after food for fifteen years, with obstinate constipation. The tongue was red and smooth—pulse frequent—epigastrium not very sensitive. "Leeches and counter-irritants were used to the epigastric region, *without much relief*, but the patient *lost all pain, the bowels were relieved*, and in a few weeks she was completely established by confining her to farinaceous and milk diet, and giving the following medicines :—

"℞. Pulv. rhœi gr. iv., morphiæ muriatis gr. $\frac{1}{12}$, M. ft. pil. ter die sumend; c. cochlear. iij. larg. misturæ sequent. ℞. Infus. cascarillæ ℥ vij., magnes. sulphatis ℥ ß, magnes. carb. pond. ʒ i ß, tinct. aloes, ℥ ss, acidi hydrocyanici ♏xv., tinct. humuli ʒ ij, M. capiat cochlear. iij. larg. ter die. These medicines acted freely, without occasioning pain or any uneasiness. They were employed by the patient for three months with the greatest benefit, occasionally increasing the quantity of morphia."

We may observe that there is some little incongruity in saying that the leeches and counter-irritation did little good, when it seems that she lost the pain. But it is probable that we do not clearly comprehend the sentence, which is rather obscure. Mr. P. informs us that he has seen numerous cases "yield almost magically to the combination he has just mentioned." We heartily wish that other practitioners, may be as fortunate. Let us just observe that the mixture quoted for the lady is not quite an eight ounce mixture, and that more than half of it was daily taken—and we cannot help thinking that seven and-a-half minims of prussic acid per diem is a pretty considerable dose to begin with. To the composition we have no objection, except that it is not always safe to establish the *habit* of giving morphia, hop, &c. three times a-day in dyspeptic affections. We know many instances where the habit could never be broken, and the patients became little less than opium eaters.

2. *Vomiting and Diarrhœa.*—" In a great majority of instances, both these symptoms are dependent on chronic irritation of the gastro-intestinal mucous membrane of the inflammatory kind." Leeches and blisters, when tenderness of the epigastrium exists, are recommended—purgatives to be avoided—and " mild opiates, antacid or absorbent remedies, as the hyd. cum creta with pulv. ipecac. comp. — a grain or two of rhubarb, with the same anodyne—and the prussic acid, to be administered."

3. *More acute forms, with great epigastric tenderness, and irritability of the vascular system.*—In these forms Mr. P. applies leeches daily in small numbers, with anodyne fomentations to the region of the stomach in the intervals—tepid gruel, or thin farinaceous food—with such medicines as the following:—

℞. Acid. nitro-muriat. ♏ xl., morphiæ muriat. gr. ½ ad gr. j., syr. simp. ℥ j., aquæ distillat. ℥ vij., cap. coch. iij. mag. 4tis horis.

These are the forms which Broussais has taken as a type of the whole series.

4. *Fulness, distention, acidity, flatulence, eructations, &c. after food.*—These are the most common symptoms of all, and it is to this group that the term " indigestion" is most frequently applied. Our author thinks that they " mark a condition of the stomach in which active hyperemia, on morbid fulness of blood, not amounting to inflammation, is the pathological character of the disease." In such cases when the stomach is empty the feelings are easy, and the mucous membrane returns to the normal condition. Now as it is the presence of food that causes the turgescence of vessels, we ask if this be not through the agency of the nerves—in other words, it is morbid sensibility of the gastric nerves, which cannot bear the contact of the usual food without pain, and without that degree of excitation which reddens the surface and fills the capillary vessels. Here we are obliged to withdraw all stimulating alimentation, and keep the patient upon the very lightest and most easily digested food. Our author, after cautioning against rough purgatives in this condition of stomach, recommends the following form of aperient:

℞. Pil. hydrarg. gr. ij.
Pulv. rhei. gr. iij.
—— ipecac. comp. gr. j.
Mucilag. acacia. q. s. ut. ft. pil ij. bis terve in die sumend.

To be taken with the cascarilla mixture already quoted. From some painful experience in this complaint, we confidently aver that few dyspectic stomachs will bear the *quantity* above prescribed, granting that the *combination* were the very best that could be imagined.

We are disposed to think that Mr. Parker has underrated the effects of acidity in the stomach. The dyspeptic is generating this acid from morning till night—and if neutralized by magnesia or other substance this hour, it will be as bad as ever the next. Till the stomach loses this most unfortunate acidifying property, it is necessary to correct the product almost every hour in the day. The effects of acidity in the stomach are not always or even generally, HEART-BURN. A host of wretched feelings, both of body and mind, are the ordinary consequences. The bulky draughts and mixtures of magnesia and bitters above described, will augment rather than relieve these feelings—and we have found that the bicarbonate of soda itself, in the form of a lozenge, is the very best form for correcting acidity. Shepherd's bicarbonate of soda lozenge; or what is fully as good, and much more reasonable, Ward's (33, Great Russell-street) bicarbonate of soda lozenge, is an admirable antacid. A single lozenge will instantly change the whole feelings of the dyspeptic sufferer, and make him quite a different person. The remedy is necessary many times a day. To Mr. Parker's advice of small relays of leeches to the epigastrium, when there is much tenderness or pain there, we have no objection, the patient being kept on milk and farinaceous diet. Mr. P. thinks that the mucilaginous and acidulated drinks, so much prescribed by the Continental physicians, are too much neglected in this country. We may here, once for all, observe that as the Continental fevers are of a very different type from those of this country;—so are the whole class of gastric complaints. And we venture to predict that our author will find out this difference, ere long, and modify his treatment accordingly. Our author winds up the work with some observations on the principal remedies used in dyspepsia. General bleeding, he observes, can never be necessary or proper, unless acute supervene on chronic gastritis. Local bleeding, on the contrary, is highly beneficial. "The large local bleedings resorted to by Broussais, and the physicians of his school, have been productive of infinite mischief." But why so? Broussais is no fool, nor an inattentive observer. And it would be exceedingly strange if he has gone on for more than a quarter of a century, doing "infinite mischief," with his eyes open! A far more natural solution of the question is to be found in the observation we made just now, namely that the diseases are different in the two countries. And why not? The diet, manners, habits, temperaments—the whole constitution, moral and physical, are totally different on the two sides of the Channel—and so are the maladies. So must also be the treatment of those maladies.

*Aperients.* These are prescribed on all occasions by some practitioners—and proscribed by others. If doctors had all suffered from dyspepsia in their own persons, they would not thus fly to extremes. They would have known that drastic purgatives are injurious—mild laxatives beneficial. The morbidly sensible nerves of the stomach and bowels will not bear strong cathartics—neither will they bear the presence of the morbid secretions, and therefore require to be daily freed from them.

"The best aperients that can be used in these diseases are combinations of the pil. hydrargyri with rhubarb or aloes, combined with the pil. galbani co., the extracts of hops, lettuce, or hyoscyamus, or the salts of morphia. Calomel, combined with the pil. aloes comp. and some sedative, is also in certain cases useful. The proportion of the mercurial for each dose should rarely exceed one grain. These remedies, with solutions of the neutral salts in bitter infusions, to which the hydrocyanic acid is added, are the forms of aperient which I have invariably found most useful; they operate freely without pain or uneasiness, and generally afford the patient very marked relief." 296.

*Sedatives.* Great importance is attached to this class of remedies by our author. "In *all* forms of inflammation, there is mostly an exalted state of the sensibility of the inflamed part."

"The peculiar organization, however, of certain nerves, particularly those of the ganglionic system, and the system of the par vagum, render the exalted sensibilities of the mucous surfaces of the stomach and intestines inappreciable by the brain, unless they pass a certain limit. Hence, in some instances, inflammatory disease of these organs proceeds to actual disorganization, without the patient being aware of its existence; whilst, in others, a slight degree of inflammation will produce intense febrile excitement. It is from a knowledge of the peculiar sensibilities of these parts that we may see the great use of sedatives in the treatment of their inflammatory or other forms of disease; and it is, also, from this circumstance, that I never prescribe an aperient remedy in diseased conditions of the stomach without combining it with some preparation of morphia, the hydrocyanic acid, or the extract of henbane. The best sedative that can be employed is the muriate of morphia. In all inflammatory affections of the stomach, this remedy, combined with aperients or with alkalies, or given merely to allay pain or irritability, is of great use. Others, however, may be given according to circumstances, such as the acetate of morphia, the hydrocyanic acid, the liq. opii sedativus, or the extracts of hop, lettuce, or henbane. These are the chief sedatives of use in such affections, and perhaps they answer all the necessary indications. They are generally more useful in combination with alkalies or aperients than when given simply and uncombined. The external application of sedative remedies to the epigastric region, in many painful affections of the stomach, I have found of very great service, whether these affections are primitive, or whether they result from organic change." 207.

*Antacids and Absorbents.* A favourite remedy of this kind is, a combination of the mist. cretæ, of the London Pharmacopæia, with "large doses of hydrocyanic acid." The ponderous carbonate of magnesia and soda are also recommended.

*Tonics.* The following extract contains a resumé of our author's remarks on this class of remedies.

"Tonics are useful in many morbid states of the stomach, which may be referred to four classes:—

1. Primitive morbid conditions, resembling inflammatory affections, which are aggravated by an antiphlogistic treatment, or by aperients.

2. States of disease, succeeding to inflammation, which have been benefited by an antiphlogistic treatment in the commencement, but where this no longer affords relief, or adds to the severity of the symptoms.

3. Various morbid states of the sensibility of the stomach. These are occasionally accompanied by intermittent neuralgic affections in other parts of the body.

4. States of general debility, and many local symptoms, as pain, nausea, and vomiting, which accompany confirmed organic diseases of the stomach." 301.

Mr. Parker has not alluded to the nitrate of silver, as a most powerful sedative in morbid sensibility of the gastric and intestinal nerves, although it is fast creeping into use even amongst the cautious German practitioners. The work is also singularly defective in tracing the *moral* causes, as well as the *moral* effects of dyspepsia—a circumstance that convinces us that the work is founded on Continental facts and foreign doctrines. Yet with these and many other defects which we could easily point out, Mr. Parker has compiled and composed one of the most valuable works on the subject of gastric disorders and diseases that has ever appeared in the English language —or, we believe, in any other tongue. We could not say less—and we need not say more.

